# QAIS-DSNN: Tumor Area Segmentation of MRI Image with Optimized Quantum Matched-Filter Technique and Deep Spiking Neural Network

**DOI:** 10.1155/2021/6653879

**Published:** 2021-01-18

**Authors:** Mohsen Ahmadi, Abbas Sharifi, Shayan Hassantabar, Saman Enayati

**Affiliations:** ^1^Department of Industrial Engineering, Urmia University of Technology (UUT), P.O. Box: 57166-419, Urmia, Iran; ^2^Department of Mechanical Engineering, Urmia University of Technology (UUT), P.O. Box: 57166-419, Urmia, Iran; ^3^Department of Electrical Engineering, Princeton University, P.O. Box: 08544, Princeton, NJ, USA; ^4^Department of Computer and Information Science, Temple University, P.O. Box: 19122, Philadelphia, USA

## Abstract

Tumor segmentation in brain MRI images is a noted process that can make the tumor easier to diagnose and lead to effective radiotherapy planning. Providing and building intelligent medical systems can be considered as an aid for physicians. In many cases, the presented methods' reliability is at a high level, and such systems are used directly. In recent decades, several methods of segmentation of various images, such as MRI, CT, and PET, have been proposed for brain tumors. Advanced brain tumor segmentation has been a challenging issue in the scientific community. The reason for this is the existence of various tumor dimensions with disproportionate boundaries in medical imaging. This research provides an optimized MRI segmentation method to diagnose tumors. It first offers a preprocessing approach to reduce noise with a new method called Quantum Matched-Filter Technique (QMFT). Then, the deep spiking neural network (DSNN) is implemented for segmentation using the conditional random field structure. However, a new algorithm called the Quantum Artificial Immune System (QAIS) is used in its SoftMax layer due to its slowness and nonsegmentation and the identification of suitable features for selection and extraction. The proposed approach, called QAIS-DSNN, has a high ability to segment and distinguish brain tumors from MRI images. The simulation results using the BraTS2018 dataset show that the accuracy of the proposed approach is 98.21%, average error-squared rate is 0.006, signal-to-noise ratio is 97.79 dB, and lesion structure criteria including the tumor nucleus are 80.15%. The improved tumor is 74.50%, and the entire tumor is 91.92%, which shows a functional advantage over similar previous methods. Also, the execution time of this method is 2.58 seconds.

## 1. Introduction

Brain tumors, which are well known to be one of the most common diseases of the nervous system, can cause many damages to human health and can also result in death. In this matter, the most common type of brain tumor among adults is glioma [1]. These tumors can be classified based on their grades as follows: Low-Grade Gliomas (LGG) exhibit benign trends and provide better patient awareness, whereas High-Grade Gliomas (HGG) are malignant, which may lead to receiving worse patient awareness [2]. The medical image of brain tumors helps assess disease development before and after treatment. Several imaging techniques, such as MRI, CT, PET, and SPECT imaging, have been used to examine brain tumors. However, MRI imaging is now the main imaging technique that can be used for glioma's diagnosis and treatment, because it has advantages such as good soft-tissue disparity, multiplied parameters, shooting in the desired direction, noninvasive photography, and so on. It also has various sequences, such as T1 weight images, T1 or T1ce-enhanced contrast, T2 weight, and Fluid Attenuation Inversion Retrieval (FLAIR). These sequences offer additional details about different parts of brain tumors [3]. For instance, the tumor area via peritumoral edema may be diagnosed in FLAIR and T2 images. Conversely, the tumor nucleus area without peritumoral edema is more prominent in images of T1 and T1ce. In this way, the different main MRI methods focus on detailed information of images, which describe the features of brain tumors under several sides.

For a medical diagnosis, accurate segmentation of these tumors is critical and needs therapeutic planning. Segmentation of brain tumors in an automatic way and existing infrastructures from medical imaging allow for accurate diagnosis of tumors. It can help plan surgery and the treatment of brain tumors by providing a more efficient and better diagnosis [4]. In particular, it is critical to divide these tumor tissues, such as enhancing core, necrosis, edema, and nonenhancing core in terms of the natural brain tissue, containing white matter (WM), gray matter (GM), and cerebrospinal fluid (CSF). Nevertheless, the precise automatic segmentation of these tumors is a challenging issue due to several reasons. In image segmentation operations, the outlines between the normal tissues and brain tumor are blurred because of the partial size effects, the gradient filtering intensity, and the magnetic field artifacts. Moreover, brain tumors are very varied in terms of size, shape, and location in patients. It is recommended to utilize a novel, robust, and fast method with the utmost care in the field of image segmentation. The segmentation of different images is a separate issue, and the right method should be designed according to each structure that should be segmented with a specific purpose. Deep convolutional neural networks have done very well in recent years in brain tumor segmentation [5]. In this regard, the convolutional neural network (so-called CNN) is a popular deep learning model that can elicit some favorite features for the original data classification [6].

This article proposes a new optimal framework of the brain tumor segmentation of MRI images that uses the structure of an optimized deep spiking network with the Quantum Artificial Immune System (QAIS). This framework is fully integrated with the QAIS-DSNN and conditional random field (CRF) combination. In the first step, a multiplied level architecture network is proposed to consider interdependence segmentation among neighboring pixels and supplementary information in various layers and measures. The background textual information of the three-dimensional MRI images is essential for brain tumor segmentation that is not taken into account by the CNNs. The study also introduces connected CRFs to correct the mapping probability attained by QAIS-DSNN.

## 2. Literature Review

To date, several methods have been proposed for MRI imaging. This section examines an overview of several classified methods.

### 2.1. Research on Deep Learning-Based Methods

The importance of MRI imaging methods for brain tumors in recent years with deep learning principles and methods due to high applications and relevant results has been highly regarded. In [7], the design of different types of convolutional neural network architecture is proposed in the form of 3 × 3 windowing with a deep layer in different grades of gliomas specimens using small nuclei. A two-way convolutional neural network model has been proposed in [8], and one channel provides detailed features of local and the other provides universal feature extraction. In [9], a convolutional neural network architecture has been created as a cascaded CNN to obtain the local dependencies of tags, achieving better performance in segmentation. Besides, they selected a two-step training strategy to address label imbalance distribution. Recently, there are advantages of multiscale features of the convolutional neural network in segmentation work [10–16]. In general, there are two methods to elicit features of multiplied scales: the first method is to use feature mapping of different network levels to show multiscale features [10].

In this respect, a multiscale convolutional neural network has been suggested to divide the retinal vein in [17]. Scale images are identified at different stages of the convolutional neural network to obtain the retinal arteries' probability mappings. Also, in [18], the structure of the Fully Convolutional Neural Network (FCNN) was developed for training with CRF; however, the process of training them was extremely time-consuming and expensive in terms of memory consumption. The second case is the transfer of versions on a different scale from the input image using the same network [10]. Also, multiscale features have been obtained by the convolutional neural network in [18]. This paper adopted three-dimensional CRFs to process segmentation results, but configuring three-dimensional CRFs is a complex process. In [18], different sizes from a convolutional neural network architecture have been used as cascaded CNN to record multiscale features. Due to this research and, of course, many other types of research that are beyond the scope of this research, the convolutional neural network has achieved significant achievements. The ability to learn neural networks with architecture and fixed parameters is limited, and the useful information, for three-dimensional MRI data, may be overlooked.

Some researchers use two-dimensional [19] or three-dimensional convolutional neural network models [18, 20, 21] to deal with three-dimensional images. For brain tumor segmentation, a three-dimensional semantic segmentation network based on the encoder-decoder architecture was developed in this way [22]. A hierarchical segmentation system that has varied the segmentation into three binary tasks has been proposed [19, 23, 24]. They also taught models of segmentation from sagittal, coronal, and axial perspectives. In the practical step, to achieve the final results, they averaged the SoftMax outputs obtained in the mentioned perspectives. Even though these methods do work very well, they raise both memory consumption and fiscal complexity. Thus, fiscal models, such as conditional random fields (CRFs) and Markov Random Fields (MRFs), are mainly employed to investigate spatial text information. In [25], a neonatal structure of a deep neural network called the Growing Deep Convolutional Neural Network (GCNN) is presented to segment MRI images to diagnose brain tumors.

There is also another method combined with GCNN that is a Stationary Wavelet Transform (SWT). The hybrid deep learning method is simulated with the use of BraTS2018 dataset and evaluated using the peak signal-to-noise ratio (PSNR), the average square error, and so on. In [26], a complete convolutional neural network with pyramidal features is presented as an Atrous convolution for brain tumor segmentation by MRI images. This research uses data sets from BraTS2013, BraTS2015, and BraTS2018, the results of which have a functional advantage over lesion structure, including tumor nucleus, improved tumor, and the whole tumor, compared to previous methods, especially the convolutional neural network. These results are based on the Dice criterion, 76.88% for the tumor nucleus, 74.43% for the optimized nucleus, and 86.58% for the entire tumor. Also, in [27], the convolutional neural network is used in three dimensions based on a method called Test-Time Augmentation. This research uses BraTS2018 data and shows the results of its evaluation with a lesion structure, including tumor nucleus, improved tumor, and whole tumor, with functional superiority over many convolutional methods and deep networks. These results were in two ways using the Dice criterion, which was 90.21% for the tumor nucleus, 79.72% for the optimized nucleus, and 85.83% for the entire tumor. In a similar study, in [28], the convolutional neural network is considered to be multicascaded (CNN) and conditional random field proposed as MCCNN. The results of this study, based on the lesion structure criteria, were 71.78% for the improved nucleus, 88.24% for the total tumors, and 74.81% for the tumor nucleus. For breast imaging monitoring and data system ranking, Kang et al. [29] indicated a dominant fuzzy full-connected layer. The aim of the model was to establish complementary scoring properties for semantic segmentation with fuzzy rules.

### 2.2. A Review of Deep Spiking Neural Network

First of all, it should be noted that sparks are the neurons of the neural network that use spikes instead of neurons in the spiking neural network, and a set of neurons in an input layer with spikes is called a spark. spiking neural networks (SNNs) are driven by the processing of biological knowledge, which communicates in parallel scattered and nonsynchronous binary signals. In neuromorphic hardware, SNNs indicate some appropriate features such as fast inference, low energy use, and event-dependent processing of information. It creates interesting applicants to apply deep learning (DL) networks effectively and a selection process for several learning tasks at a computer. Here, SNNs consider a wide range of training methods, including the conversion of convolutional deep networks to SNNs, limited preconversion training, and a variety of biological motivations [30].

**Figure 1:**
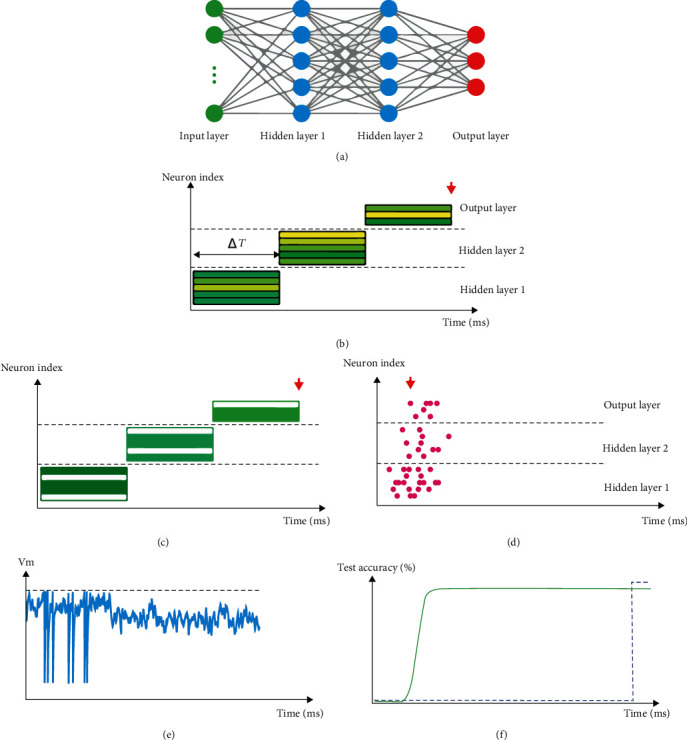
General structure and mechanism of a spiking neural network or SNN [30].

Neural networks are usually read if they have at least two hidden layers of nonlinear input conversion. In this study, only feedback networks are considered to calculate mapping from input to output. Spiking neural networks were initially studied as biological information processing models in which neurons exchange information through spikes. Here, all spikes are expected to be stereotypical events; in this way, data processing is minimized to two main factors: First of all, the timing of spikes, for example, firing frequency, the relative timing of pre-/postsynapse spikes, and special patterns of movement. Secondly, the identification of the synapses used means it is possible to connect nerve cells, whether the synapse is stimulating or inhibitory. With regard to the degree of detail of the simulation neurons, the two neurons are the point at which the input spikes alter their (somatic) membrane potential immediately or are built together with complex (dendritic) spatial structures as multichamber models. Hence, the dendritic currents will communicate before that. There were also changes to physical capacity. Here, several models of spike neurons, such as Hodgkin's Huxley model, integrate-and-fire, and spike response, explain the evolution of membrane potential and the spike of different rates of detail in development. Essentially, the membrane potential of the stream merges with the entry of the spikes and generates a new spike since the threshold is crossed. After the spike is obtained, the small axon is sent to all the linked nerve cells by a delay via the axon, based on which the membrane potential is adjusted to a certain base.  Figure 1 shows this.

Direct communication between spiking and analog neural networks is formed by assuming a stable state, by considering the activation of an analog neuron is equal to the firing rate of a spiking neuron. Many geometric models used those rate codes to describe brain computational processes. Nevertheless, more complex processes can also form the neural spike models, which depend on some reference signals or relative timing between spikes, such as network fluctuations. Temporary codes are very important in biology; even a spike or small time-consuming changes in neuron firing may cause different reactions, as most decisions must be calculated before a reliable estimation of the spike [30].

In addition to the biological definition of SNNs, they contain a pragmatic functional representation in the field of neural engineering; SNNs are commonly referred to as spikes and are event-based. An event here is a collection of digital information defined by a time marker's origin and destination address. Unlike biologically motivated SNNs, it may have several bits of load information. The source for this protocol is the address index or AER (Address Event Representation) protocol, which is used after processing to link to event-based sensors through digital connection to neural chips or digital hardware. Event-based visual sensors use the loading bit to differentiate between silent and visual events; however, the loading bit can also be used to send other types of information to postsynapse targets, potentially to calculate more advanced functions than the fire integration method or integrate and fire used. The reason for researching SNNs is that, in real-world activities, the brains display considerable cognitive function. With continuing efforts to enhance our perception of brain-like calculations, models closer to biology are closer to achieving human intelligence than more abstract, or at least more computationally effective, models [30].

In this way, SNN methods are ideally appropriate to process the space-time information based on neuro sensors, which are themselves energy-efficient. Sensors collect precise environmental information, and SNNs can use some useful time codes for their calculations. This information processing is also the focus of the event, which is denoted whenever a small amount of information is not recorded in the SNN; it does not do much calculation, but the SSN creates more spikes when an activity explosion is recorded. It leads to a very efficient way of calculation, assuming that information from the outside world is usually scattered. Also, time-domain input is another precious piece of information compared to framework-based approaches, where an artificial timeline is introduced entered by the sensor. It can result in an effective calculation features such as optical current or stereo inequality combined with spike-sensitive learning rules. In deep SNNs, asynchronous axis-based computing mode results in the rapid dissemination of prominent information through multiple network layers. In practical terms, SNNs must be run on neuromorphic hardware to take advantage of this effect. This process is a quasisimultaneous data processing combined with an event-based sensor, which implies that after the first input spikes are registered, the first estimated output of the final layer is immediately available. Also, for multilayered networks, it is right as the spikes extend immediately to the higher layers as soon as enough activity is generated by the bottom layer. You do not have to wait to complete of the complete input series, which is unlike traditional deep neural networks, where it is important to completely charge all layers until the final output is calculable. The primary performance spikes are inevitably based on incomplete data. It was thus concluded that deep SNNs would increase their efficiency in classification and decrease the processing time of the spike more than their input. To decrease the expected delays in inference, SNNs can also be specifically fitted. SNNs are the computational model chosen to run highly energy-efficient neuromorphic hardware devices supporting a data-driven processing mode and maintaining local calculations, thus prohibiting access to expensive memory [30].

Here, despite recent advances, one of the major deep SNN disadvantages is their accuracy in standard metrics such as MNIST, CIFAR, or ImageNet which is not as good as that of their machine learning counterparts. The existence of the benchmarks present in traditional frame-based images can perhaps be attributed to this, to some extent. A sort of conversion of the picture to the Spark sequence that is typically inefficient is therefore required. The lack of training algorithms that take advantage of Spark neurons' features, such as efficient timescales, is another limiting factor. In contrast, several approaches employ many approximations according to the rate of use of convolutional deep learning neural networks, denoting that no progress can be expected. Deep SNNs may be practical in these cases and maybe faster, in which they get more efficient than convolutional systems, where SNN runs on diagonal neural hardware. For SNNs, the training algorithms are difficult to analyze due to their noncomputational and discontinuous computational methods, which generated direct use of successful techniques behind the scenes, especially for deep neural networks be difficult [30]. In traditional AI standards, the performance of SNNs should only be considered as concept proof, but not as the ultimate research goal. If biology is the model of spike networks, it can be concluded that they are designed for behavioral tasks such as making decisions based on continuous current input when moving in the real world. Whereas brains may solve these things, they are certainly not optimal for it. Recently, the Internet environment lacks good metrics and evaluation metrics that can measure effective performance in the real world [30].

## 3. Proposed Approach

The preprocessing phase of the proposed approach is aimed at reducing the initial noise. In the following, the operation of segmentation and extraction of features is aimed at distinguishing tumor masses from the data set. The preprocessing section applies a method called Quantum Matched-Filter Technique, followed by a CRF-based QAIS-DSNN combination approach.

### 3.1. Preprocessing Phase

Initially, there will be a preprocessing phase involving noise reduction. Every single image is displayed in a combination of local threshold and active contouring using a two-dimensional array of pixels; their values are integers in the range of [0,255]. Local thresholds initialize images in two steps. First, the input noise image is considered the primary image to which image noise removal will be applied. This operation is mainly utilized as a local search operator to enhance the initial images, using the Quantum Matched-Filter Technique (QMFT). The use of local thresholds and active contours has been used in this paper because they are computationally faster than other methods in the literature. Thus, at the end of the first step, there will be a decomposed image. In the second step, thresholding is done on the detail coefficients, and one of these decomposed sections is randomly selected and sent to a reconstruction operation. The reconstruction section can be defined:

- *Gaussian blur*: uses a Gaussian filter to filter the image. Between 3 × 3 pixels and 5 × 5 pixels, the filter size is accidentally selected
- *Mean filter (averaging filter)*: filters the image using an average filter
- *Intensity change*: all image pixels are multiplied by a similar criterion randomly selected in the range [0.7, 1.3]
- Implement light-intensive sections in quantum and reverse processing that performs the QMFT

Then, the following operations are performed:

- *One-point row*: a pixel row is chosen randomly
- *One-point column*: it is identical to the preceding form, but instead of a row, the column is considered
- *Point-to-point random*: accidentally, every pixel is selected from the decomposition until a new image is created
- Identify all points in a row and column in the image to reduce the majority of noise as QMFT

After analysis, when the selected range value [0.1] is less than the local search rate in the QMFT, a new image of the local search operator may pass. As the decomposition is complete, the entire image is sorted by its pixel value. Then the best aspect ratio in the image is considered as a quantum value in the sequel. A signal in MRI images may be broken down into multiple displaced or resized displays of features known at the feature extraction stage. Local thresholds and active contours can be used to analyze an image into its components. It is possible to perform image segmentation operations after applying QMFT along with local and active contouring thresholds. In this case, the local threshold coefficients and the active contour based on QMFT can be destroyed to eliminate some details. Local thresholds and QMFT-based active contours have a tremendous advantage in separating fine detail in an image. Active contour can be used to isolate very fine details of an image. At the same time, local thresholds can detect large details, combining fine and large details, and reading all rows and columns linearly and diagonally. Quantum satisfies QMFT to minimize the noise in the MRI image. QMFT based on local thresholds and active contours can create a sparse display. A local and active contouring threshold function with QMFT has two main features, the first of which is a function of oscillation or wave appearance, such as

(1) $$\begin{array}{c} {\left.\int_{-\infty }^{0}\Psi \left(t\right)\right|}^{2}dt<\infty . \end{array}$$

In this case, most of the energy in *Ψ* (t) is limited to a limited time, which is in the form of

(2) $$\begin{array}{c} \int_{-\infty }^{0}\Psi \left(t\right)dt=0. \end{array}$$

The proposed method is generally calculated to reduce the noise in

(3) $$\begin{array}{c} \Omega \left(I\right)=\left(\underset{\Omega }{\sum }\sqrt{1+\beta^{2}{\left|\nabla I\right|}^{2}}\right)+\frac{\lambda }{2}{\left(I-I_{0}\right)}^{2}. \end{array}$$

In Equation (3), the term (*I* − *I*_0_)^2^ ensures a certain degree of validity and accuracy between the rated and original image, in which *I* denotes the rated image while *I*_0_ means the noisy image. The ∇*I* parameter is defined as the sum of the variable adjustment periods, *β* and *λ* are the balancing parameters, and *Ω* is the sum of the image's points. The purpose of minimizing Equation (3) is to decrease total image diversity while maintaining accuracy and validity. The balancing values are changed from 1 to the size of the image for both *β* and *λ* to minimize Equation (3).

### 3.2. Segmentation with QAIS-DSNN Combined Approach

The deep spiking neural network presented in this study, due to its high flexibility, can use a linear and nonlinear functions such as sigmoid or sinusoidal in hidden layers. Use nonderivative as well as intermittent activation. By default, DSNN has

(4) $$\begin{array}{c} y\left(p\right)=\overset{m}{\underset{j=1}{\sum }}\beta_{i}\beta_{j}g\left(\overset{n}{\underset{i=1}{\sum }}w_{i,j}x_{i}+b_{j}\right). \end{array}$$

According to Equation (4), *β*_*i*_ displays the weights between the input and the hidden layers, and *β*_*j*_ displays the weights between the output and the input layers (*b*_*j*_). The value of the neuron threshold is in the hidden layer or the bias. *g*(.) is an activator or stimulus function. The weights of the input layer, *w* (*i*, *j*), and bias, *b*_*j*_, are randomly assigned. The beginning of the neuron number on the input layer *n* and the neuron number on the hidden layer *m* is assigned to activation function *g*(⋯). If the known parameters in the general equilibrium are combined and controlled on the basis of this information, the output layer will be similar to

(5) $$\begin{array}{c} H\left(w_{i,j},b_{j},x_{i}\right)=\left[\begin{array}{c} g\left(w_{1,1}x_{1}+b_{1}\right)\\ g\left(w_{n,1}x_{n}+b_{1}\right) \end{array}\begin{array}{c} \cdots \\ \cdots \end{array}\;\begin{array}{c} g\left(w_{1,m}x_{m}+b_{m}\right)\\ g\left(w_{n,m}x_{m}+b_{m}\right) \end{array}\right],y=H\beta . \end{array}$$

The main goal is to minimize errors as much as possible in all models of training-based algorithms. The *y*_*p*_ the output error function is obtained by the actual *y*_main_ output in DSNN, which can be done with two training sections, ∑_*k*_^*s*^(*y*_main_ − *y*_*p*_) and the test section, ‖∑_*k*_^*s*^(*y*_main_ − *y*_*p*_)^2^‖. The output *y*_*p*_ generated by the real output, *y*_main_, must be identical with the same *y*_*p*_ for both functions. An unknown parameter is specified when this equation is performed and the results are satisfying. While spikes have been used to understand local label dependencies, for medical images such as MRI, they are not appropriate. Typically, that is because anatomical forms have complex shapes for models that are distinct.

Moreover, either the temporal or the spatial relationship of MRI data also plays a critical role in classification, which should be paid attention to with regard to the method. Therefore, it is better to modify the mapping of the probability achieved by DSNN. A rather low-probability matrix may be the *H*-matrix, which means that the amount of data in the training process will not be identical to the total number of data characteristics. But it would be a big challenge to reverse [*H*] and find weights or *β*. A fully connected CRF matrix is used to overcome this challenge in DSNN, which can develop an approximate reversal of the matrix that cannot be reversed. It can reduce the size, selection, and extraction of features at the segmentation with high precision and incredible speed compared to other methods. Currently, CRFs have been implemented in many medical imaging applications because they perform well when modeling some complex spatial data dependencies. In this way, to segment brain tumors, CRFs can be used not only to model the relationship between an image pixel and poster properties but also to make local pixel properties and their labels dependent. As discussed earlier, in [11] and [26], CRFs were employed to visualize images through image formulation as neural networks. Nevertheless, the process of training their method is cumbersome and mathematically complex. In contrast, CRFs will be utilized as a suitable hash method. Using the fully connected matrix and CRF layer, the output matrix *β*^∗^ and the matrix *H*^∗^ are all inverted and generalized by *H*. Therefore, due to the improvement of DSNN as CRF-DSNN in this section, the problem of the output weights in DSNN has been resolved and converted to *B*^∗^ = *H*^∗^. In general, CRF-DSNN becomes a series of repeating units over time in the training phase. CRF-DSNN will be able to act as a belt conveyor and add or subtract information to neurons. Unlike deep learning structures and other classification models, such as backup conveyor machines or nanoscale works, no weight update is performed during training. CRF-DSNN can define features at the segmentation. By reducing CRF energy performance, a suitable model is taught that can be modeled as

(6) $$\begin{array}{c} E\left(Y\right)=\overset{N}{\underset{i}{\sum }}\Psi_{u}\left(y_{i}\right)+\overset{N}{\underset{\forall i,j,i\neq j}{\sum }}\Psi_{p}\left(y_{i},y_{j}\right), \end{array}$$

where *u*, *p* ∈ {1, 2, ⋯, *C*_*n*_) are the designations of the segmentation and *i*, *j* ∈ {1, 2, ⋯, *N*} properties are specific pixels of the original image or *I*. Ψ_*p*_(*y*_*i*_) = −log*P*(*y*_*i*_ | *I*) is the negative logarithmic probability where *P*(*y*_*i*_ | *I*) is a probability obtained by DSNN per pixel *i*. While measuring the capabilities of a matrix pair of CRFs in a fully connected layer, it deals with the relationship between each pixel that is defined as

(7) $$\begin{array}{c} \Psi_{p}\left(y_{i},y_{j}\right)=\mu \left(y_{i},y_{j}\right)\overset{M}{\underset{m=1}{\sum }}w^{\left(m\right)}k^{\left(m\right)}\left(f_{i},f_{j}\right), \end{array}$$

where *M* = 2, the number of Gaussian nuclei and *w*^(*m*)^ indicate a weight for the Gaussian nucleus mth, and *μ*(*y*_*i*_, *y*_*j*_) = [*y*_*i*_ ≠ *y*_*j*_] is the label of consistent function. *k*^(1)^ displays the core appearance, which tries to assign the same class labels to neighboring and adjacent pixels with the same intensity. *k*^(2)^ displays the kernel smoothness, which is associated with eliminating unnecessary areas. These two steps are shown as

(8) $$\begin{array}{c} k^{\left(1\right)}\left(f_{i},f_{j}\right)=\exp\left(-\frac{\left|s_{i}-s_{j}\right|}{2\theta_{\alpha }^{2}}-\frac{\left|e_{i}-e_{j}\right|}{2\theta_{\beta }^{2}}\right), \end{array}$$

(9) $$\begin{array}{c} k^{\left(2\right)}\left(f_{i},f_{j}\right)=\exp\left(-\frac{\left|s_{i}-s_{j}\right|}{2\theta_{\gamma }^{2}}\right). \end{array}$$

*e*_*i*_ and *e*_*j*_ are the light intensities of the pixel *i* and *j* and *s*_*i*_ and *s*_*j*_ are the corresponding spatial coordinates. *f*_*i*_ and *f*_*j*_ mean the characteristics of each pixel pair, i.e., the brightness intensity and spatial information. *θ*_*α*_, *θ*_*β*_, and *θ*_*γ*_ show the parameters of the Gaussian nucleus, respectively. However, some points in the mass may not be segmented in this way, so this algorithm optimization will be done in layers. In general, the DSNN method's layers are the use of the input layer with the number of neurons (spikes). Then, the structure of the training and testing layer used convolution, pooling, and fully connected layers along with CRF. Then, a SoftMax layer is embedded for it and then an output layer to display the work. The training layer window is in the form of a matrix, 9 × 9 in the convolution layer, 7 × 7 in the pooling layer, and 5 × 5 in the maximum section (Maxpool). The structure of the fully connected layer is 9 × 9. The SoftMax layer is also 7 × 7.

The Quantum Artificial Immune System (QAIS) is used to optimize the segmentation process during high-altitude neural network training in the SoftMax layer section. The QAIS uses a factor called an antigen. In an MRI image, all antigens are detected through a memory-based adult detection system, which has a fault tolerance experiment with a choice of the colon and immune mutations. Colonial choices and immune mutations are the other two factors of the QAIS algorithm. The more MRI data, the more copies are duplicated. In this algorithm, reproduction is plural, especially like a crossover in the genetic algorithm [12, 13]. Antibodies focus on modern quantum memory detection systems in mass segmentation in real time and examine detection and cross-sectional states against the MRI image structure.

The display of MRI image data is performed by a set of antigens Ag = {ad | ad ⊂ *S*}, in which the antigens determine the ad. They display one bit of binary string bits' properties that are represented by MRI image data antigens. These bits contain Trait codes. Also, *S* is the spatial state in the QAIS that is presented by *S* = {0, 1}^*l*^ and displays all the activities of the primary population in the image in segmentation. *l* is the natural state number in the QAIS algorithm, which is considered as a constant value. There are two states of self-adjusting and non-self-adjusting in the artificial immune system algorithm. The self-adjusting state (self ⊂ Ag) displays all MRI image data and the non-self-adjusting state (nonself ⊂ Ag) displays all the segmented data. Therefore, there is a relationship between self-adjusting and non-self-adjusting states, represented by self ∪ nonself = Ag and self∩nonself = ∅ equations.

The safety diagnostic set is also *D* = {ab, *p*, *t*, age, cnt| ab ∈ *S*, *p* ∈ *R*, *t*, age, cnt ∈ *N*}, where ab is the antibody, *p* is the concentration of the antibody, *t* is the tolerance of error, age indicates the age of memory and the maturity of genes, *R* is a set of real numbers, and *N* is the case number natural genes. Memory detection set *M*_*d*_ = {*d* | *d* ∈ *D*, *d* · cnt > *β*} and the gene recognition maturity group are shown as *T*_*d*_ = {*d* |*d* ∈ *D*, *d* · age < *λ*, *d* · cnt < *β*}. There is also immaturity, which is defined as the immaturity of genes that are expressed as *I*_*d*_ = {*d*|*d* ∈ *D*, *d*.*t* < *α*}. In these relationships, *D* = *M*_*d*_ ∪ *T*_*d*_ ∪ *I*_*d*_, where *α* represents the threshold for error in detecting immature status, *λ* represents the gene life cycle, and *β* represents the threshold value for detecting gene maturity.

In order to establish and evaluate the structure of diagnostic development, an immature gene detector becomes the mature state detector, which will be successful in the fault tolerance phase. When the adaptive time between the adult gene detector in the gene's life cycle and the antigens activated exceeds the *β* threshold, the adult detector clones or collects itself and then evolves into a memory detector. It means that genes and antigens will have a memory. Once the antigens are recognized by a specialist, he/she assembles the mature diagnostic compound. To ensure that antigens are effectively detected and that a variety of antibodies are detected in the reagent (mature or immature), they will detect known or unknown attacks. A total of three operators are used for the QAIS algorithm to improve the transverse distribution of MRI image data, which includes dependency assessment, reproduction selection, or safety and mutation combination, which are described separately.

Hamming distance is used to compute the correlation for antigen detection. For example, the error tolerance mode is considered to create a model of correlation assessment. An unsuccessful identifier can succeed, if the immature identifier has never been compared to all elements of the self-organizing group in the *α* variable. On the contrary, it can lead to the death of genes and antigens. The *s* ∈ self is assumed, and Equation (10) shows how id is determined by the *s*.

(10) $$\begin{array}{c} f_{\text{match}}\left(s,\text{id}\right)=\left\{\begin{array}{cc} 1, & \frac{f_{\text{affinity}}}{l_{d}}>\gamma ,\\ 0, & \text{otherwise}. \end{array}\right. \end{array}$$

According to Equation (10), 1, 0 indicate whether the id is compatible with *s* and *l*_*d*_ is the size of the id detector, so *f*_affinity_ is used to calculate the correlation between s and id. Likewise, *γ* as *γ*(0 ≤ *γ* ≤ 1) represents the correlation threshold. Equation (11) is used to implement the mature error detector of the immature id, and Equation (12) is used to add the self-enforced identifier time when the results of the Equation (11) return to 1, and if *t* ≥ *α*, the immature identity must develop into a mature

(11) $$\begin{array}{c} f_{\text{tolerance}}\left(s,\text{id}\right)=\left\{\begin{array}{cc} 0, & \exists s\in \text{self},f_{\text{match}}\left(s,\text{id}\right)=1,\\ 1, & \text{otherwise}, \end{array}\right. \end{array}$$

(12) $$\begin{array}{c} \left\{\begin{array}{c} T_{d}=T_{d}\cup \text{id},I_{d}=I_{d}-\text{id},\text{if id}\in I_{d},\text{id}\cdot t\geq \alpha ,\\ \text{id}\cdot t=\text{id}\cdot t+1,\text{if id}\cdot t<\alpha \wedge f_{\text{tolerance}}\left(s,\text{id}\right)=1. \end{array}\right. \end{array}$$

The colonial or combination choice operator performs cellular operators in mature and memory diagnosis. Equation (13) is used to detect cloning state and a mixture of genes and antigens.

(13) $$\begin{array}{c} C_{\text{num}}\left(d\right)=\left\lceil \xi \cdot \left(1-\frac{n_{d}}{N_{d}}\right)\right\rceil . \end{array}$$

According to Equation (13), *ξ* (>0) is a colonial or combination constant. *N*_*d*_ = *T*_*d*_ ∪ *M*_*d*_ shows all the combinations. The colonial determinant or combination factor is used to analyze the performance of cellular operators in mature and memory diagnosis. Equation (13) is used to detect the cloning state and a mixture of genes and antigens. In Equation (14), *T*_cln_ and *M*_cln_ display the colonic selection group or group of memory and mature detectors. After making a colonial selection or group of genes and antigens in a generation, the cloned or combined section is added to the adult diagnostic group, and the same detector *d*_*t*_(∈*T*_*d*_), in the colonial selection group, or the *T*_cln_ and *M*_cln_ combination will be removed.

(14) $$\begin{array}{c} T_{d}=T_{d}\cup T_{\text{cln}}\cup M_{\text{cln}}-\left\{d_{t}\;\right|\;\left.d_{t}\in T_{\text{cln}}\vee d_{t}\in M_{\text{cln}}\right). \end{array}$$

The goal of the immune mutation operator is to enhance the detector's diversity with the mutation of the antibody generation in the corresponding detector, which is used to improve the ability to detect antigens. Considering the (*l*_*d*_ − *f*_affinity_(*d*, ag)) bit, the *d*(∈*N*_*d*_) detector set is matched by the ag(∈Ag) antigen; these bits are used by 0.1 instead of randomly. *l*_*d*_ displays the size of *d*. The mutant detector is used as an immature detector by the self-regulating set. To detect the adult mode, if the adaptive time is greater than the activated threshold *β*, the stimulus operation is performed using Equation (13) and then combined with the memory detector according to

(15) $$\begin{array}{c} M_{d}=M_{d}\cup \left\{d\;|\;d\in T_{d},d\cdot p=\eta_{1},d\cdot \text{age}=0\right\}. \end{array}$$

Equation (15) is assumed to represent the arranged numbers of the reagent that can be matched with antigens. Therefore, the memory diagnosis segment is combined with Equation (16), but this occurs when the memory diagnosis segment can be successfully matched with antigens.

(16) $$\begin{array}{c} M_{d}=\left\{d\;|\;d\in M_{d},d\cdot p\left(t\right)=\eta_{1}+\eta_{2}\cdot d\cdot p\left(t-1\right),d\cdot \text{age}=0\right\}. \end{array}$$

Equation (17) also illustrates a different type of antigen removed in the MRI image data for display.

(17) $$\begin{array}{c} d\cdot p=\left\{\begin{array}{cc} d\cdot p\;\left(1-\frac{1}{\theta -d\cdot \text{age}}\right), & d\cdot \text{age}++<\theta ,\\ 0, & d\cdot age++\geq \theta . \end{array}\right. \end{array}$$

Intensity and variety are two important features of swarm intelligence algorithms. The intensity is in the search of the best-obtained solutions and choosing the best candidate points. It is worthwhile to mention that the diversification procedure can allow the optimizer to explore the search space more efficiently. Inertial weight parameters (*w*_*n*_, *w*_*f*_) indicate changes in optimal global attractiveness that affect the convergence rate and update each mass's position in the combination algorithm QAIS-DSNN. In the proposed QAIS-DSNN hybrid algorithm, the inertial weights (*w*_*n*_, *w*_*f*_) are set to a large value to emphasize exploration, i.e., 0.9, which are set in the initial search mode, finally reduced to 0.1 linearly for the importance of linear optimization. Inspired by the classic artificial immune system, it is guaranteed that quantitatively, global characteristics for optimal segmentation can be determined when using the spiking neural network. As the number of repetitions increases, the initial population is encouraged to local search. Finally, the population should only carefully search for a local area without discovery to find out if there are any other masses. As a result, the first quantum combination strategy is to provide a linear weight reduction of the new frequency. The model (*w*_*n*_, *w*_*f*_) is created as

(18) $$\begin{array}{c} w_{\text{total}}=0.9-\left(\frac{0.9-0.1}{\text{MI}}\right)\times J, \end{array}$$

where [0.1, 0.9] is the inertial weight range and MI is the maximum number of repetitions, in which *J* denotes the number of repetitions. As such, both *w*_*n*_ and *w*_*f*_ are linearly decreased from 0.9 to 0.1 in the repeat cycle. The developed mixed QAIS-DSNN algorithm may be trapped in local improvement due to the presence of different iterative cycles in the tumor fractionation improvement process, in addition to the high research capacity. Therefore, to solve this problem, it is possible to provide a comparative update strategy for the *C*^best^ parameter that is best to assist neurons and primary residents of the proposed algorithm out of the optimal local areas. In this strategy, *C*^best^ is considered the best at a great value in the initial phase of finding the optimum value of the QAIS-DSNN algorithm with strong exploration ability (global search) and gradually decreasing with increasing frequency for accurate searching.

**Figure 2:**
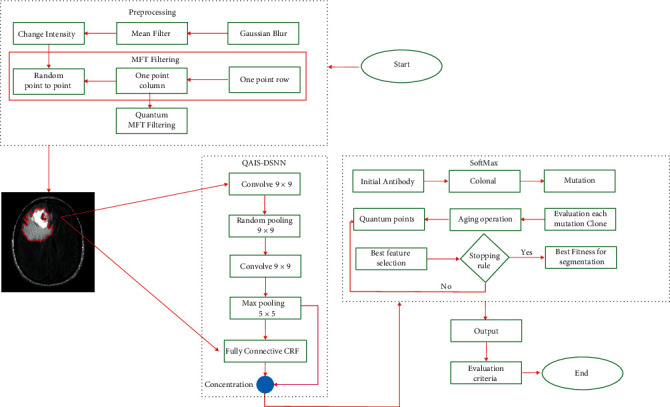
Flowchart of the proposed approach.

Equation (19) displays the optimal value for the better adaptive update scheme *C*^best^ used here.

(19) $$\begin{array}{c} C^{\text{best}}=2\times 1-\frac{J}{\text{MI}}, \end{array}$$

where *J* means the number of repetitions, whereas MI denotes the maximum number of repetitions. The next step is to introduce a novel method for updating the neurons and the initial population to accelerate global convergence. Initially, the status vector is updated by

(20) $$\begin{array}{c} \Delta t=C_{t}\overset{\text{NV}}{\underset{j-1}{\sum }}\left(\text{U}{\text{B}}_{j}-\text{L}{\text{B}}_{j}\right), \end{array}$$

where NV is the total number of variables, UB_*j*_ is the upper limit, and LB_*j*_ is the lower limit of the variable in the *j*^th^ variable. *C*_*t*_ is the search environment, the same as the main input image. Randomization is then performed that prevents trapping in the optimal local solution, so randomization is introduced in Equation (21) with the value of *α*_rand_, which is a randomization parameter.

(21) $$\begin{array}{c} X_{i}\left(t+\Delta t\right)=X_{i}\left(t+\Delta t\right)+\left(X_{\text{gbets}}-X_{i}\left(t+\Delta t\right)\right)+\alpha_{\mathrm{rand}}\times \left(\mathrm{rand}-\frac{1}{2}\right), \end{array}$$

where *X*_gbets_ is the position of each neuron, and the initial population of the combination approach and rand is a random number generated, represented as a uniform distribution in the range of [0, 1]. In general, the flowchart of the proposed approach is shown in Figure 2.

### 3.3. Investigating the Computational Complexity of the QAIS-DSNN Method

Here, the computational complexity of the developed QAIS-DSNN algorithm is investigated. Computational complexity includes temporal and spatial complexity. The time complexity of the QAIS-DSNN algorithm depends on two steps including calculation of the motion and updating of the positions of the neurons and the initial population. Therefore, the complexity of time can be defined in

(22) $$\begin{array}{c} O\left(\text{QAIS}‐\text{DSNN}\right)=O\left(t\left(O\left(\text{intensity neighbor edges}\right)+O\left(\text{position update}\right)\right)\right), \end{array}$$

(23) $$\begin{array}{c} O\left(\text{QAIS}‐\text{DSNN}\right)=O\left(t\left(n^{2}\times d+n\times d\right)\right)=O\left(tn^{2}d+tnx\right)=O\left(tn^{2}d\right), \end{array}$$

where *t* is the maximum repetition cycle, *n* denotes the number of neurons or initial population, and *d* is the dimensions of the problem.

## 4. Simulation and Results

**Figure 3:**
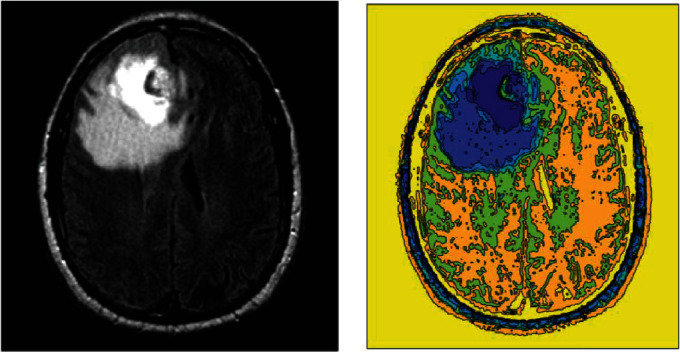
Input image.

BraTS data is a collection of brain tumor MRI images, including 145 folders for patients under different conditions. The dataset consists of 4 versions from 2012 to 2018. Database versions are getting better every year. The primary data is in DICOM format which have been converted to JPEG format for easier use through DICOM Viewer software. The input images are three-dimensional. Due to the large size of the images in the BraTS, we used 1000 video input samples to study the proposed approach. The simulation will be done in MATLAB 2015b environment and a system with 7-core processor specifications with 6 MB of cache and 3.6 MHz and 6 GB of memory in Windows 10. When the simulation is performed, all BraTS2018 data are trained and tested by the proposed method. For visualization, an example of images is shown to examine the proposed approach's results, step by step. Initially, the input image is given to the system, as shown in Figure 3.

**Figure 4:**
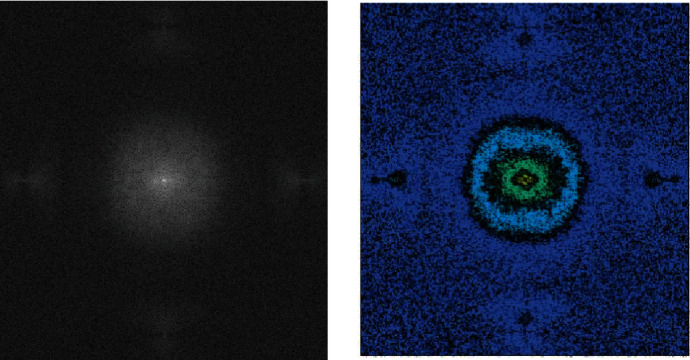
QMFT noise reduction algorithm applied in a row, column, and diagonal without repetition.

**Figure 5:**
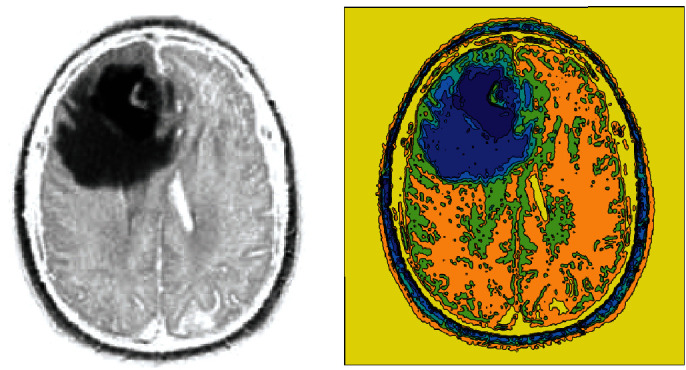
The result of image noise reduction and highlighting.

In all BraTS2018 data images, an initial noise reduction is required, using the QMFT algorithm, in which the image is read linearly, columnar, and diagonally without any repetition to reduce noise. The schematic of this output is in the form of Figure 4, and the result of the image that the noise reduction operation and its initial highlighting is in Figure 5.

**Figure 6:**
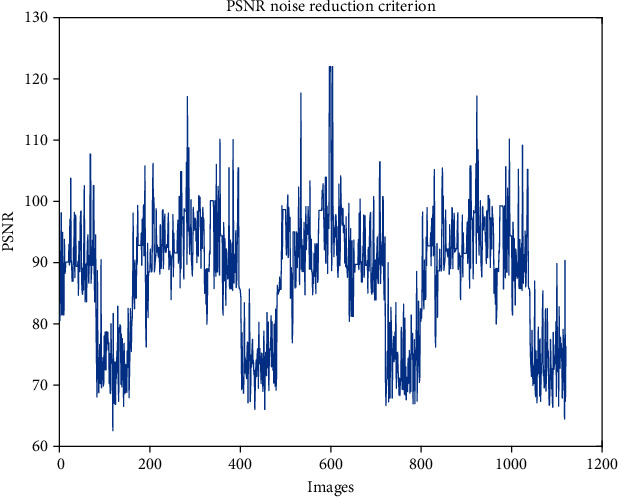
The PSNR criterion for noise reduction measurement.

The value of peak signal-to-noise ratio (PSNR) is illustrated in Figure 6. The analysis is done for 1120 MRI images. The mean value of PSNR is 87.35. It can be seen that the noise reduction provides an interesting picture in which a good segmentation can be applied. For this purpose, a deep neural network spiking or DSNN method is applied to the noise reduction operation output, and the BraTS2018 video data set is trained, which will be 75% training and 25% test. But the combined DSNN approach with the QAIS algorithm is made in this section so that the overall result is visible. According to the DSNN structure, it is observed that five input layers are considered, in which all BraTS2018 video data are placed. Then, there are three rows of training layers, the first of which is the training deep layer. In this row, from the deep layer, one by one, the convolution layer with 9 × 9 windowing, and then the random polarizing layer with 7 × 7 windowing, again the convolution layer with 9 × 9 windowing, and then the maximum polarization layer as 5 × 5 is located. The stimulus function of this layer is a zygomatic logarithm in that the number of general layers is 20. Then, the fully connected layer is associated with CRF, which is considered as 10 layers.

**Figure 7:**
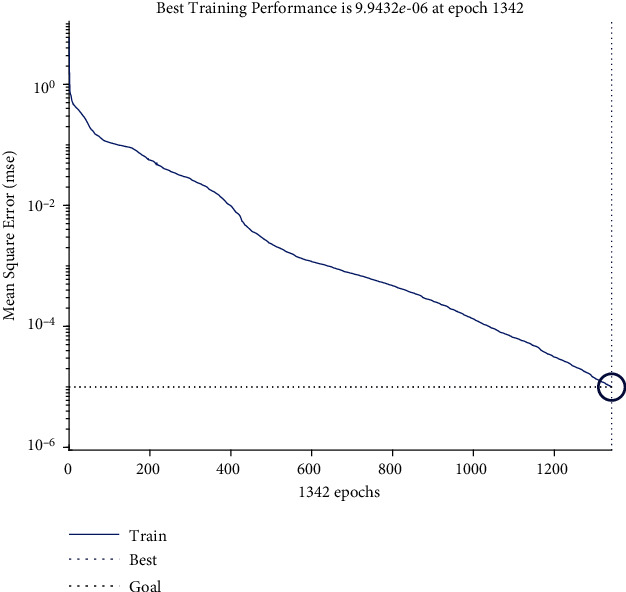
Mean square error of the training process for 1342 epochs.

**Figure 8:**
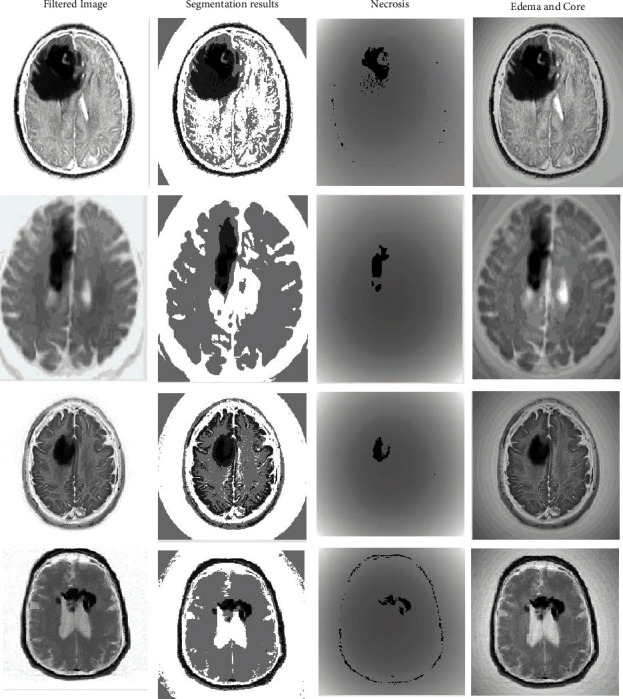
Display of training operations to test the segmentation of the image and identify the masses. From left to right: the overall result of noise reduction as input in QAIS-DSNN and QAIS-DSNN approach segmentation, finding necrosis, and finding edema (part completely black) and nucleus (the almost white part inside the edema).

Then, there is a SoftMax layer with the QAIS algorithm designed to optimize DSNN segmentation during training and testing, more accurate mass detection, and feature selection operations. Its drive function is linear. There are separate settings for the QAIS algorithm. The initial population of this algorithm is considered to be 200. The colonial rate is 0.04, and its repetition rate is 10 cycles for optimizing the DSNN algorithm segmentation and selecting features in the SoftMax layer. In the end, there is an output layer that is a layer to display the output. The number of raw data training and testing rounds in QAIS-DSNN is equal to 7000 rounds. The QAIS-DSNN core is resilient back-propagation, and its performance is measurable with average error squares. The training process is illustrated in Figure 7. The termination criterion for the training process is mean square error as 10^−5^. Regrading Figure 7, 1342 epochs lead to converge the training process. When the proposed approach is applied to multiple images, the overall result will be Figure 8.

**Figure 9:**
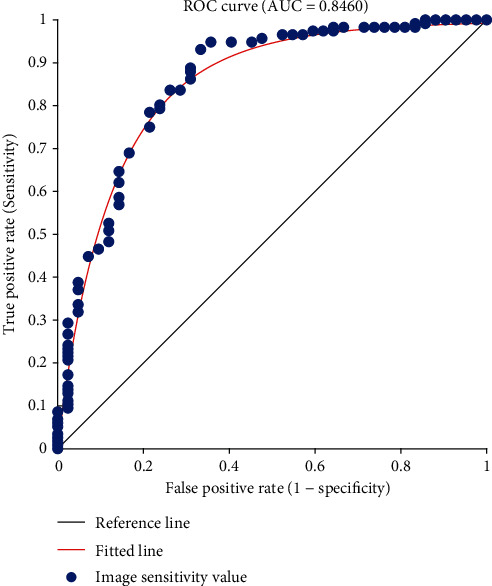
ROC diagram and AUC rate.

The ROC chart and the AUC rate are the proposed approach in Figure 9. This curve is known as one of the most important evaluation criteria, which measures the efficiency of classification operations in a system. In general, in a binary classification system in which the differentiation threshold differs, the ROC curve is a graphical representation of the degree of sensitivity or correct prediction versus false prediction. The ROC curve is also shown by plotting the correct positives against the predicted false positives. A number which measures and evaluates an aspect of performance is the area below the ROC curve. This area below the curve is called the AUC. A value above 0.7 to 1 indicates an excellent level of prediction and classification performance. According to Figure 9, it is observed that the value of AUC is a number below one, which shows the optimization of the proposed approach as much as possible. The presence of some similar sections with cancerous masses in the available data and presented method led to the creation of a series of minor errors that have not been adapted to the fitting line. The blue circles are the criterion values, and the red line is the ROC diagram on which the data is fitted. In some areas where the data is a bit far away, an error occurs and leads to a decrease in inaccuracy. Also in the middle line is regression called the ROC peak relative to regression, and the area below it is AUC. After applying the proposed approach, it is necessary to compare the proposed approach with other proposed methods, which are examined in terms of different evaluation criteria to determine the guarantee of the proposed approach. For this purpose, Table 1 shows a comparison in terms of average error squares, signal-to-noise ratio, and accuracy. Also, a comparison has been made in terms of Dice evaluation criteria for tumor nucleus, total tumor, and tumor areas, the results of which can be seen in Table 2.

The next comparison is the percentage-based accuracy for segmentation to distinguish the mass region from the images, which are averaged from the BraTS data set. The results are reported in Table 3.

Finally, a comparison is made in terms of computational complexity in terms of time between the method presented in this study and the methods in reference [25], the results of which are shown in Table 4. It is noteworthy that this study has listed the system used during the processing of the proposed method, and this comparison is made on a case-by-case basis with reference [25].

Based on the results of the comparisons in terms of evaluation, it is observed that the proposed approach is optimal in terms of the mean error squares of most methods, but the GCNN [25] and BAT-IT2 FCM [38] algorithms have better results than the research approach. In terms of signal-to-noise ratio, the proposed method of compared algorithms has had better results. In terms of Dice evaluation criteria, most research is on the same level. There are differences in the parts of the whole tumor, the tumor nucleus, or the improved part of the tumor, depending on the different methods available. In terms of accuracy, the prediction approach has better results, but with the GCNN [25] algorithm, it is 0.01% more efficient. Also, the results were obtained at the level of convolutional methods, and the computational complexity of the proposed approach has been implemented in the system; however, the computational complexity can be seen by combining the existing algorithms.

## 5. Conclusion

This article is innovative in the field of noise reduction and segmentation of MRI images to detect the area of tumor masses. Also, we used the QMFT method to find noise and reconstruct it with adjacent pixels to process them horizontally, vertically, and diagonally. It is formed in the fastest time and has been able to move the noise by identifying and reviewing neighbors and matching the pixel data with neighbors based on the edge of the image. Then, the segmentation operation was performed with a QAIS-DSNN combination approach. In this approach, the deep neural network of spiking with CRF is considered, so that after the input layer—including neurons (spikes)—the training layer has convolution and polarizing layers. All of them are connected to the CRF format. Then, there is a SoftMax layer outside the training layer, which is optimized for segmentation and detection to accurately identify tumor features, in this method with the QAIS. The simulation results show that the proposed QAIS-DSNN approach has a functional advantage over the previous methods evaluation criteria. Among these evaluation results, we can point out the accuracy in segmentation and detection of the exact mass area in MRI images with an accuracy of 98.21%. Also, the average rate of error squares is 0.006, and the peak rate of the signal-to-noise ratio is 97.79 decibels. The use of lesion structural criteria includes a tumor nucleus of 80.15%, improved tumor of 74.50%, and a total tumor of 91.92%, which is a functional advantage over similar previous methods. Reducing computational complexity compared to previous methods and improving execution time by 2.58 seconds also confirms it. In the future, the plan is to use a huge dataset or transfer this system to the breast, lung, and some other tumor detection tasks. Moreover, we are going to add this automated segmentation method for CNN-based segmentation ground truth images. Furthermore, the presented filtering system can be added as a layer in the CNN method and change the resolution of the matrix in each iteration.

## Figures and Tables

**Table 1:** The comparison of the proposed approach with previous ones in terms of mean square error, peak signal-to-noise ratio, and accuracy.

The peak signal-to-noise ratio (decibels)	Mean square error	Method	Reference
72.1	0.23	KNN	Mittal et al. [25]
88.4	0.045	Genetic algorithm	Mittal et al. [25]
74.2	0.021	SVM	Mittal et al. [25]
73.5	0.256	SOM	Mittal et al. [25]
94.2	0.012	CNN	Mittal et al. [25]
96.64	0.001	GCNN	Mittal et al. [25]
77.7	0.001	BAT-IT2FCM	Alagarsamy et al. [31]
97.79	0.006	QIAS-DSNN	Proposed approach

**Table 2:** The comparison of the proposed approach with previous ones in terms of Dice evaluation criteria.

Tumor improvement or ET areas	Tumor nucleus or TC	Total tumor or WT	Method	Reference
81.84%	88.34%	91.2%	ADNN-PSO	Irfan Sharif et al. [32]
85.83%	79.72%	90.21%	3D cascaded CNN-TTA	Wang et al. [27]
79.19%	85.40%	90.31%	Cascaded CNN	Wang et al. [27]
77.07%	73.04%	89.56%	Multiclass WNet+TTA	Wang et al. [27]
71.78%	74.81%	88.24%	MCCNN	Hu et al. [28]
72.29%	76.75%	86.23%	Two-stage	Zhou et al. [26]
70.9%	75.1%	85.1%	Ordinary fusion	Zhou et al. [26]
73.44%	76.58%	86.38%	3D UNet	Zhou et al. [26]
72.55%	75%	84.94%	APFNet	Zhou et al. [26]
74.43%	76.88%	86.56%	APF+3D-CRF	Zhou et al. [26]
74.50%	80.15%	91.92%	QAIS-DSNN	Proposed approach

**Table 3:** Comparison of the proposed approach with previous methods in terms of accuracy in terms of percentage.

Accuracy (%)	Method	Reference
98.20%	GCNN	Mittal et al. [25]
95%	3D cascaded CNN-TTA	Wang et al. [27]
88.50%	MCCNN	Hu et al. [28]
96.12%	BAT-IT2FCM	Alagarsamy et al. [31]
92%	ADNN-PSO	Irfan Sharif et al. [32]
98%	PSO-LDA-GA-ANN	Sharif et al. [33]
98.21%	QAIS-DSNN	Proposed approach

**Table 4:** Comparison of the proposed approach with previous methods in terms of computational complexity over time.

Computational complexity in terms of time (seconds)	Method	Reference
0.84 seconds	GCNN	Mittal et al. [25]
1.6 seconds	CNN	Mittal et al. [25]
3.2 seconds	Genetic algorithm	Mittal et al. [25]
2.7 seconds	SVM	Mittal et al. [25]
4.2 seconds	SOM	Mittal et al. [25]
3.8 seconds	KNN	Mittal et al. [25]
2.58 seconds	QAIS-DSNN	Proposed approach

## Data Availability

The data that support the findings of this study are openly available in BraTS2013, BraTS2015, and BraTS2018 at https://www.med.upenn.edu/sbia/brats2018/data.html
